# Delayed Diagnosis of Rhino‐Orbito‐Facial Mucormycosis in a Patient With Newly Diagnosed Diabetes and Diabetic Ketoacidosis: A Successful Case From a Resource‐Limited Setting

**DOI:** 10.1155/crdi/3763579

**Published:** 2026-09-02

**Authors:** Filmon Tesfay Riesehaymanot, Ruth Ghirmay Gebregziabiher, Haben Dawit Tewelde, Meron Asheber Gebremicael, Meron Berhane Tesfay

**Affiliations:** ^1^ Internal Medicine Department, Keren Zonal Referral Hospital, Keren, Eritrea; ^2^ Gynecology and Obstetrics Department, Orotta College of Medicine and Health Sciences, Asmara, Eritrea; ^3^ Pathology Department, Orotta College of Medicine and Health Sciences, Asmara, Eritrea; ^4^ Internal Medicine Department, Teseney Hospital, Teseney, Eritrea; ^5^ Emergency Department, Barentu Zonal Referral Hospital, Barentu, Eritrea

**Keywords:** conventional amphotericin B, debridement, diabetes mellitus, diabetic ketoacidosis, mucormycosis, resource-limited settings

## Abstract

**Background:**

Rhino‐orbito‐facial mucormycosis is a rapidly progressive, life‐threatening fungal infection that predominantly affects patients with uncontrolled diabetes mellitus, particularly those with diabetic ketoacidosis (DKA). Early diagnosis remains challenging in resource‐limited settings because initial manifestations often mimic bacterial sinusitis.

**Case Presentation:**

We report a 38‐year‐old woman with newly diagnosed diabetes mellitus presenting with left facial pain, swelling, and nasal discharge accompanied by mild DKA. She was initially treated for bacterial maxillary sinusitis with antibiotics and insulin therapy; however, progressive facial necrosis and subsequent orbital involvement prompted surgical debridement and histopathological examination, which confirmed mucormycosis. The patient underwent repeated surgical debridement, conventional amphotericin B therapy, and intensive glycemic control, resulting in complete clinical recovery without recurrence during 3 years of follow‐up.

**Conclusion:**

This case highlights the diagnostic challenges of mucormycosis in resource‐limited settings and emphasizes the importance of maintaining a high index of suspicion in diabetic patients whose facial or orbital symptoms worsen despite appropriate antibacterial therapy. Early histopathological confirmation, prompt antifungal treatment, and multidisciplinary management remain essential for improving outcomes, even when advanced diagnostic resources are limited.

## 1. Introduction

Mucormycosis is a rare but life‐threatening invasive fungal infection caused by fungi of the order Mucorales. It predominantly affects immunocompromised individuals, especially patients with uncontrolled diabetes mellitus, hematologic malignancies, or those receiving immunosuppressive therapy [1]. Among its clinical forms, rhino‐orbito‐facial mucormycosis is common and aggressive, typically beginning as sinusitis and rapidly progressing to facial and orbital involvement. If left untreated, it can extend intracranially, progressing to rhino‐orbito‐cerebral mucormycosis (ROCM) which carries even higher morbidity and mortality [1–3]. Diabetes mellitus, particularly when complicated by ketoacidosis, impairs host immune defenses by altering neutrophil function and providing an acidic, hyperglycemic environment conducive to fungal proliferation [1, 4, 5].

Early diagnosis of mucormycosis is challenging due to nonspecific symptoms overlapping with bacterial sinusitis or orbital cellulitis, which may delay initiation of appropriate antifungal therapy [6]. Imaging modalities such as MRI and CT help delineate the extent of infection, but diagnostic access is often limited in low resource settings. Histopathological confirmation of broad, ribbon‐like, nonseptate hyphae invading tissue remains the gold standard [7]. Management involves urgent surgical debridement combined with systemic antifungal therapy, most commonly liposomal amphotericin B, alongside aggressive control of underlying metabolic abnormalities [8].

Despite advances in medical therapy, mucormycosis continues to carry a poor prognosis. Infection confined to the sinuses and facial structures tends to have a relatively better outcome; in an explicit comparison, mortality rates for cases that progress to full ROCM range from 25% to 62% [1]. These challenges are amplified in resource‐limited settings, where both diagnostic tools and antifungal agents may be scarce [9]. We report a case of delayed rhino‐orbito‐facial mucormycosis in a woman with newly diagnosed diabetes mellitus and mild DKA who was successfully managed with repeated surgical debridement, conventional amphotericin B, and multidisciplinary care despite significant diagnostic and therapeutic challenges.

## 2. Case Presentation

A 38‐year‐old Eritrean woman presented to the emergency department with a 10‐day history of persistent left‐sided facial pain localized to the maxillary region, accompanied by left‐sided nasal discharge and progressive swelling over the left nasolabial fold. The swelling had markedly worsened during the preceding 3 days. She also reported a 1‐month history of polyuria, polydipsia, polyphagia, and significant unintentional weight loss. She denied fever, cough, visual disturbance, vomiting, diarrhea, trauma, or focal neurological symptoms. Her medical history was unremarkable, with no known history of diabetes mellitus or other chronic illnesses. She had no previous hospital admissions or surgeries and was not taking any regular medications. There was no family history of diabetes mellitus or other significant medical conditions. She denied smoking tobacco, alcohol consumption, and illicit drug use.

On presentation, the patient appeared acutely ill but was alert and fully oriented. Vital signs were as follows: blood pressure 120/80 mmHg, pulse rate 91 beats/min, respiratory rate 23 breaths/min, SpO_2_ 98% on room air, and temperature 36.1°C. Point‐of‐care blood glucose measurement demonstrated marked hyperglycemia. The head and neck examination revealed diffuse swelling over the left nasolabial fold with obliteration of the nasolabial crease and mild deviation of the mouth to the right. The overlying skin was erythematous and tender on palpation, particularly over the left maxillary sinus. Purulent left‐sided nasal discharge was present. Ophthalmological examination was initially unremarkable, with normal visual acuity, intact extraocular movements, and no evidence of proptosis. Cardiovascular, respiratory, abdominal, and neurological examinations were otherwise normal.

The initial clinical impression was acute left maxillary sinusitis with possible facial abscess in a patient with newly diagnosed diabetes mellitus. She was admitted for further diagnostic evaluation and management, including blood workup, imaging, and initiation of appropriate medical therapy. Initial laboratory investigations showed leukocytosis with a white blood cell count of 11,790/μL and neutrophilia (77.98%), hemoglobin 14.4 g/dL, and platelet count 276,000/μL. Serum electrolytes, liver function tests including alkaline aminotransferase and aspartate aminotransferase, and renal function tests were within normal limits. Urinalysis revealed marked glucosuria (+4) and ketonuria (+2). The erythrocyte sedimentation rate was elevated at 60 mm/hr, and C‐reactive protein was positive, indicating active inflammation. Glycated hemoglobin (HbA1c) was markedly elevated at 14%, indicating longstanding undiagnosed hyperglycemia.

**FIGURE 1:**
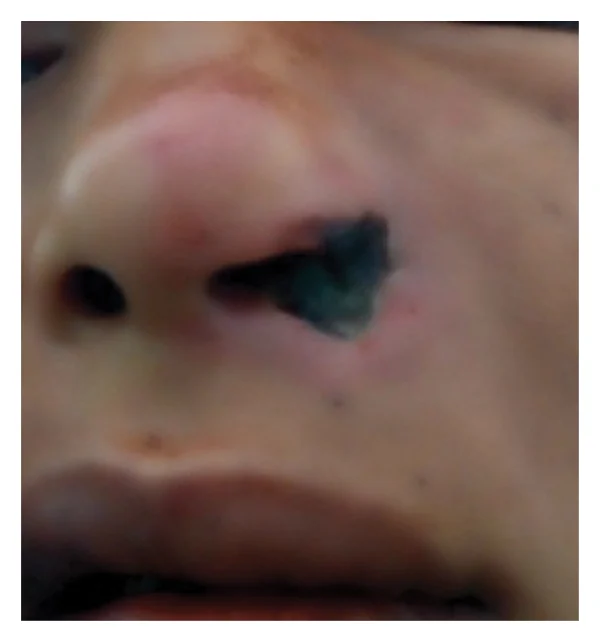
Clinical photograph (frontal view) demonstrating progressive necrosis of the left nasolabial region with surrounding erythema and soft‐tissue swelling on the seventh day of hospitalization. The development of dusky discoloration and necrotic tissue despite appropriate antibacterial therapy raised clinical suspicion for invasive fungal infection.

**FIGURE 2:**
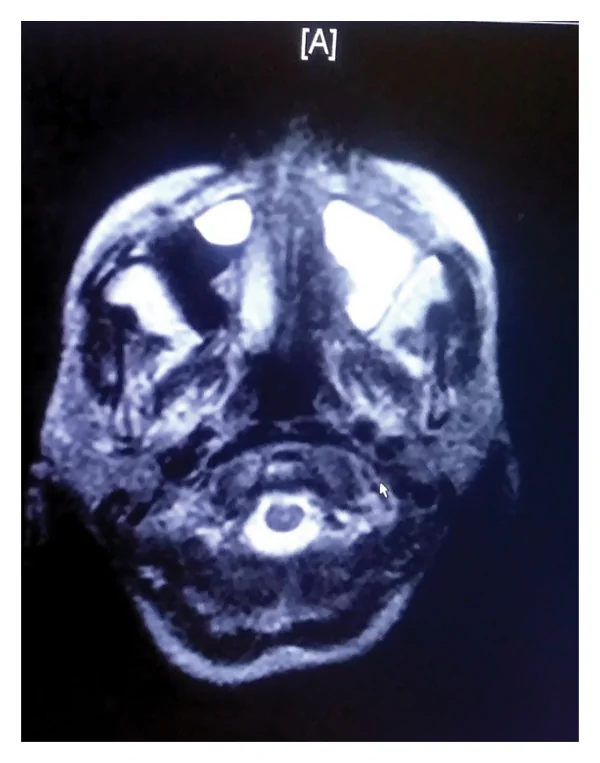
Coronal T2‐weighted magnetic resonance imaging (MRI) of the paranasal sinuses (anterior orientation indicated by [A] at top) demonstrating homogeneous hyperintense fluid within the left maxillary sinus, consistent with inflammatory sinus disease. No definite radiological features of invasive fungal sinusitis, orbital extension, or intracranial involvement were identified at the time of imaging.

**FIGURE 3:**
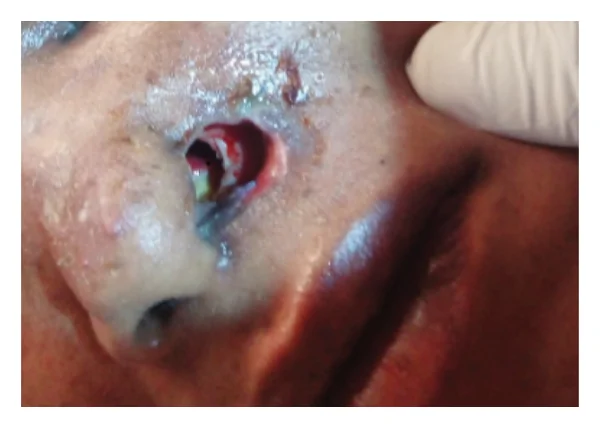
Close‐up clinical photograph (left oblique view) obtained after initial surgical debridement showing a sinus tract communicating with the left maxillary sinus through the left nasolabial fold. Progressive tissue necrosis necessitated repeated surgical debridement and further diagnostic evaluation.

**FIGURE 4:**
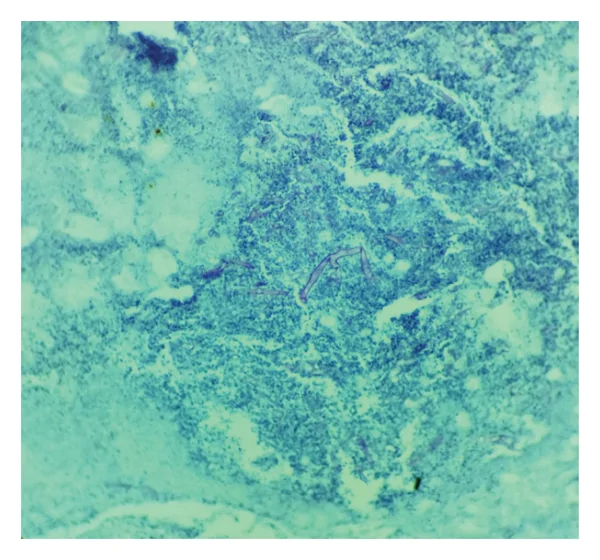
Histopathological examination of debrided tissue (hematoxylin and eosin stain, × 400 magnification) demonstrating broad, ribbon‐like, pauciseptate fungal hyphae with tissue invasion, consistent with mucormycosis and confirming the diagnosis.

The patient was admitted to the intensive care unit with newly diagnosed diabetes mellitus complicated by mild DKA and presumed bacterial maxillary sinusitis. At presentation, the patient had no clinical features strongly suggestive of invasive fungal infection, such as facial necrosis, black eschar, orbital involvement, or cranial nerve deficits. Consequently, bacterial sinusitis was considered the most likely diagnosis, although the presence of DKA placed her at increased risk for opportunistic fungal infection. Management included intravenous ceftriaxone and clindamycin, insulin infusion, intravenous fluids, and potassium replacement. Otorhinolaryngology (ENT) consultation recommended continuation of conservative management. Following resolution of DKA within 48 h, insulin therapy was transitioned to a split‐dose regimen. Despite 5 days of appropriate antibiotic therapy, the patient continued to experience severe left maxillary pain, and glycemic control remained difficult despite increasing insulin requirements. On the seventh hospital day, the left nasolabial swelling became dusky with the development of necrotic skin changes (Figure 1), raising concern for an invasive process. Magnetic resonance imaging (MRI) of the brain and paranasal sinuses demonstrated homogeneous T2‐hyperintense fluid within the left maxillary sinus without definite imaging features suggestive of invasive fungal sinusitis. There was no evidence of intracranial extension, and the orbital structures appeared preserved (Figure 2). Contrast‐enhanced CT was considered but left due to temporal unavailability in the setting. Because of progressive clinical deterioration despite appropriate antibacterial therapy, surgical debridement was performed, and tissue samples were obtained for a histopathological examination (Figure 3). Cloxacillin was added while awaiting pathological diagnosis, and daily wound care was continued with sterile saline and gauze. Five days after the initial debridement, the patient developed blurred vision, left orbital pain, progressive orbital swelling, mild proptosis, conjunctival injection, ophthalmoplegia, and reduced visual acuity. Ophthalmology consultation recommended continued broad‐spectrum antibiotics for presumed orbital cellulitis and evaluation for fungal infection. Histopathological examination demonstrated broad, ribbon‐like, pauciseptate fungal hyphae with tissue invasion, confirming mucormycosis (Figure 4). Conventional amphotericin B was initiated at 1 mg/kg/day because liposomal amphotericin B was unavailable in our setting. The patient subsequently underwent repeated surgical debridement together with daily wound care and intensive glycemic management.

Following initiation of conventional amphotericin B therapy and repeated surgical debridement, the patient’s clinical condition improved progressively. Glycemic control gradually stabilized, allowing transfer from the intensive care unit to the general medical ward. During the subsequent 2 weeks, healthy granulation tissue developed, the facial wound healed progressively, and the sinus tract closed. Serial monitoring of renal function, liver function tests, serum electrolytes, and complete blood counts remained within normal limits throughout amphotericin B therapy, with no evidence of significant treatment‐related toxicity.

Conventional amphotericin B was continued for 1 month, accompanied by meticulous wound care and optimization of insulin therapy. Before discharge, the patient received comprehensive education regarding insulin administration, blood glucose monitoring, diabetic foot care, and the importance of regular follow‐up. She was discharged on a split‐dose insulin regimen and scheduled for follow‐up in the endocrinology and otorhinolaryngology clinics.

At subsequent follow‐up visits, the patient demonstrated progressive clinical recovery without neurological or ophthalmological sequelae. The facial wound healed completely, leaving only a residual scar over the left nasolabial fold. During 3 years of follow‐up, she maintained good glycemic control with insulin therapy and experienced no evidence of recurrent mucormycosis. The patient expressed gratitude for the coordinated multidisciplinary care that preserved her vision and avoided more extensive surgical procedures. The chronological sequence of the patient’s clinical presentation, investigations, therapeutic interventions, and follow‐up is summarized in Table 1.

**TABLE 1:** Timeline of clinical events.

Time	Clinical event
1 month before admission	Polyuria, polydipsia, polyphagia, and significant weight loss.
10 days before admission	Left‐sided facial pain and nasal discharge developed.
3 days before admission	Progressive swelling over the left nasolabial fold.
Day 0	Hospital admission. Diagnosed with newly diagnosed diabetes mellitus complicated by mild diabetic ketoacidosis and presumed acute bacterial maxillary sinusitis. Intravenous fluids, insulin infusion, ceftriaxone, and clindamycin were initiated.
Day 2	Diabetic ketoacidosis resolved, and insulin therapy was transitioned to a split‐dose regimen.
Day 5	Persistent facial pain and poor glycemic control despite antibiotic therapy.
Day 7	Left nasolabial swelling became dusky with necrotic skin changes. MRI performed, followed by surgical debridement.
Day 12	Blurred vision, orbital pain, proptosis, conjunctival injection, ophthalmoplegia, and reduced visual acuity developed. Histopathology confirmed mucormycosis. Conventional amphotericin B therapy was initiated.
Following weeks	Repeated surgical debridement, daily wound care, and optimization of glycemic control resulted in progressive clinical improvement.
1 month	Conventional amphotericin B therapy completed.
At discharge	Wound healing was satisfactory. The patient was discharged on insulin therapy with endocrinology and ENT follow‐up.
3‐year follow‐up	No recurrence of mucormycosis, good glycemic control, preserved vision, and only a residual facial scar.

## 3. Discussion

Mucormycosis is an increasingly recognized opportunistic fungal infection with high mortality, particularly in patients with uncontrolled diabetes mellitus. In the present case, newly diagnosed diabetes mellitus with severe hyperglycemia (HbA1c 14%) and mild diabetic ketoacidosis (DKA) created a favorable metabolic environment for fungal invasion. Diabetes mellitus is recognized as the predominant risk factor worldwide, contributing to nearly 50% of mucormycosis cases [3, 9]. Ketoacidosis creates a favorable environment for fungal growth by increasing serum‐free iron levels, which facilitate fungal proliferation and impair neutrophil chemotaxis and phagocytosis [4].

One of the major challenges in this case was the delayed diagnosis. At presentation, the patient had facial pain, nasal discharge, and swelling without orbital involvement, black eschar, or cranial nerve deficits. These nonspecific findings were clinically compatible with acute bacterial sinusitis, making the initial diagnosis reasonable. However, persistence of symptoms despite appropriate antibacterial therapy, increasing insulin requirements, and subsequent development of facial necrosis and orbital manifestations were important warning signs that prompted reconsideration of the diagnosis. Early manifestations of mucormycosis frequently overlap with bacterial sinusitis and orbital cellulitis, resulting in delayed diagnosis and treatment [6].

Diagnosis of mucormycosis remains particularly challenging in resource‐limited settings. Although MRI and CT are valuable for determining the extent of disease, early imaging findings may be nonspecific and cannot reliably exclude invasive fungal infection. In our patient, MRI demonstrated inflammatory changes within the maxillary sinus without classical radiological features suggestive of mucormycosis. The histopathological examination ultimately confirmed the diagnosis by demonstrating broad, ribbon‐like, nonseptate fungal hyphae invading necrotic tissue, consistent with the current diagnostic gold standard [7].

Jeong et al. demonstrated that earlier tissue diagnosis is associated with significantly improved survival, whereas delays in diagnosis substantially increase mortality [10, 11]. In our patient, histopathological confirmation was obtained only after progressive clinical deterioration despite antibacterial therapy. This case, therefore, emphasizes the importance of maintaining a high index of suspicion and obtaining an early tissue biopsy whenever diabetic patients with presumed bacterial sinusitis fail to improve with appropriate treatment.

Successful management of rhino‐orbito‐facial mucormycosis requires a multidisciplinary approach involving prompt surgical debridement, systemic antifungal therapy, correction of metabolic abnormalities, and close collaboration among internal medicine, otorhinolaryngology, ophthalmology, and pathology teams. Combined medical and surgical treatment has consistently been associated with better survival than antifungal therapy alone [8, 10, 12].

Liposomal amphotericin B is currently recommended as the preferred antifungal agent because of its improved tissue penetration and lower nephrotoxicity [8, 10]. However, in many resource‐limited settings, including our institution, liposomal amphotericin B is unavailable. Conventional amphotericin B, therefore, represented the only feasible treatment option. Careful monitoring of renal function, liver function tests, and serum electrolytes allowed successful completion of therapy without significant treatment‐related toxicity.

Another important lesson from this case is that persistent hyperglycemia despite escalating insulin therapy may reflect ongoing invasive fungal infection rather than inadequate diabetic management alone. Active infection contributes to insulin resistance and stress hyperglycemia, making glycemic control difficult until the underlying infection is adequately treated [13, 14].

The major strength of this report is the detailed documentation of successful management of delayed rhino‐orbito‐facial mucormycosis in a resource‐limited setting with excellent long‐term clinical outcome after 3 years of follow‐up. The principal limitation is the absence of fungal culture and molecular identification, as diagnosis relied on the histopathological examination. Furthermore, advanced diagnostic modalities and liposomal amphotericin B were unavailable, reflecting the challenges commonly encountered in low‐resource settings.

Overall, this case demonstrates that delayed diagnosis does not inevitably result in poor outcomes. Early recognition of clinical deterioration, timely histopathological confirmation, repeated surgical debridement, prompt amphotericin B therapy, aggressive glycemic control, and multidisciplinary management remain the cornerstones of successful treatment, particularly in resource‐limited settings [8, 10].

## 4. Conclusion

Rhino‐orbito‐facial mucormycosis should be considered in patients with uncontrolled or newly diagnosed diabetes mellitus, particularly those with DKA, who present with facial pain, swelling, or orbital symptoms that fail to improve despite appropriate antibacterial therapy. This case highlights the diagnostic challenges encountered in resource‐limited settings and emphasizes that delayed diagnosis does not necessarily preclude a favorable outcome. Early clinical suspicion, prompt histopathological confirmation, repeated surgical debridement, timely initiation of amphotericin B therapy, aggressive glycemic control, and multidisciplinary management are essential for improving patient outcomes. Increased awareness among clinicians may facilitate earlier recognition and reduce the morbidity and mortality associated with this aggressive fungal infection.

## Author Contributions

F.T.R.: conceptualization, data collection, writing original draft, and reviewing the manuscript. R.G.G.: clinical management, data collection, conceptualization, and revising the manuscript. H.D.T.: clinical management, data collection, and revising the manuscript. M.A.G. and M.B.T.: clinical management and revising the manuscript.

## Funding

The authors received no financial support for this study.

## Disclosure

All authors have read and agreed to the published version of the manuscript.

## Ethics Statement

Obtaining ethical clearance from our Ministry of Health’s Ethical Clearance and Protocol Review Committee is not required for case reports.

## Consent

Written informed consent was obtained from the patient for publication of this case report and utilization of any accompanying images.

## Conflicts of Interest

The authors declare no conflicts of interest.

## Supporting Information

Additional supporting information can be found online in the Supporting Information section.

## Supporting information


**Supporting Information** Care checklist‐Mucormycosis.

## Data Availability

The data that support the findings of this study are available from the corresponding author upon reasonable request.
