# Supplementary material for: Delayed Diagnosis of Rhino‐Orbito‐Facial Mucormycosis in a Patient With Newly Diagnosed Diabetes and Diabetic Ketoacidosis: A Successful Case From a Resource‐Limited Setting
Source: Case Rep Infect Dis. 2026 Sep 2;2026:3763579. doi: 10.1155/crdi/3763579 (PMC13535859; doi:10.1155/crdi/3763579)
Supplement: Supplementary file 1 [file CRDI-2026-3763579-s001.pdf]

CARE case report guidelines

CARE Checklist of information to include when writing a case report

(cc) BY-NC-ND

Topic	Item	Checklist item description	Reported on Line
Title	1	The diagnosis or intervention of primary focus followed by the words "case report"	1-3
Key Words	2	2 to 5 key words that identify diagnoses or interventions in this case report, including "case report"	42-43
Abstract (no references)	3a	Introduction: What is unique about this case and what does it add to the scientific literature?	23-26
	3b	Main symptoms and/or important clinical findings	27-29
	3c	The main diagnoses, therapeutic interventions, and outcomes	29-34
	3d	Conclusion—What is the main "take-away" lesson(s) from this case?	35-40
Introduction	4	One or two paragraphs summarizing why this case is unique (**may include references**)	44-68
Patient Information	5a	De-identified patient specific information.	70-71
	5b	Primary concerns and symptoms of the patient.	70-74
	5c	Medical, family, and psycho-social history including relevant genetic information	75-79
	5d	Relevant past interventions with outcomes	76-77
Clinical Findings	6	Describe significant physical examination (PE) and important clinical findings.	80-89
Timeline	7	Historical and current information from this episode of care organized as a timeline	166
Diagnostic Assessment	8a	Diagnostic testing (such as PE, laboratory testing, imaging, surveys).	82-83,93-100,114-117,126-127
	8b	Diagnostic challenges (such as access to testing, financial, or cultural)	117-118
	8c	Diagnosis (including other diagnoses considered)	90-91,101-105,126-127
	8d	Prognosis (such as staging in oncology) where applicable	N/A
Therapeutic Intervention	9a	Types of therapeutic intervention (such as pharmacologic, surgical, preventive, self-care)	105-107,118-120,127-129
	9b	Administration of therapeutic intervention (such as dosage, strength, duration)	128,153-154
	9c	Changes in therapeutic intervention (with rationale)	108-110,120-121,127-128
Follow-up and Outcomes	10a	Clinician and patient-assessed outcomes (if available)	147-152,159-162
	10b	Important follow-up diagnostic and other test results	150-152
	10c	Intervention adherence and tolerability (How was this assessed?)	151-152,155-157
	10d	Adverse and unanticipated events	111-113,122-125
Discussion	11a	A scientific discussion of the strengths AND limitations associated with this case report	212-217
	11b	Discussion of the relevant medical literature **with references**.	169-211
	11c	The scientific rationale for any conclusions (including assessment of possible causes)	170-177,185-190
	11d	The primary "take-away" lessons of this case report (without references) in a one paragraph conclusion	225-234
Patient Perspective	12	The patient should share their perspective in one to two paragraphs on the treatment(s) they received	163-164
Informed Consent	13	Did the patient give informed consent? Please provide if requested	Yes ☑ No ☐
